# Exosomal miR-16-5p as a target for malignant mesothelioma

**DOI:** 10.1038/s41598-019-48133-0

**Published:** 2019-08-12

**Authors:** Phillip B. Munson, Elizabeth M. Hall, Nicholas H. Farina, Harvey I. Pass, Arti Shukla

**Affiliations:** 1 Department of Pathology and Laboratory Medicine, University of Vermont, College of Medicine, Burlington, VT 05405 USA; 2 Department of Biochemistry, University of Vermont, College of Medicine, Burlington, VT 05405 USA; 3 University of Vermont Cancer Center, University of Vermont, College of Medicine, Burlington, VT 05405 USA; 4 Department of Cardiothoracic Surgery, NYU Langone Medical Center, 530 First Avenue, 9V New York, New York, 10016 USA

**Keywords:** miRNAs, Mesothelioma, Mechanisms of disease

## Abstract

Malignant mesothelioma (MM) is an asbestos-induced cancer arising on the mesothelial surface of organ cavities. MM is essentially incurable without a means of early diagnosis and no successful standard of care. These facts indicate a deep chasm of knowledge that needs to be filled. Our group recently delved into MM tumor biology from the perspective of exosome-contained microRNAs (miRNAs). We discovered that the most abundant miRNAs in MM cancer exosomes were tumor suppressors, particularly miR-16-5p. This observation lead us to hypothesize that MM cells preferentially secreted tumor-suppressor miRNAs via exosomes. Through separate avenues of potential therapeutic advance, we embarked on an innovative strategy to kill MM tumor cells. We employed small molecule inhibitors to block exosome secretion, thereby reducing miR-16-5p exosome loss and replenishing cellular miR-16-5p leading to reduced tumorigenic capacity and miR-16-5p target oncoproteins CCND1 and BCL2. Additionally, we force-fed MM tumor exosomes back to MM tumor cells, which led to cell death, and a reduction in the same oncoproteins. We recapitulated these results with direct transfection of miR-16-5p, confirmed that this is a cancer-cell specific effect, and elucidated a part of the miR-16-5p mechanism of exosome loading.

## Introduction

Malignant mesothelioma (MM) is a remarkably deadly cancer arising after exposure to asbestos fibers^1^. Median life-span after diagnosis is 6-12 months, there is a latency period of 20-50 years after initial asbestos exposure, and MM is relatively un-diagnosable until the disease is in advanced stages^2^. Due to limited knowledge of biomarkers for asbestos exposure and early detection of this cancer, coupled with no successful therapeutic regimens other than chemotherapeutic intervention with cisplatin and pemetrexed, this disease signifies large gaps in scientific knowledge that, when filled, would greatly benefit human health^3^.

An exciting realm of cancer research has developed over the past decade by focusing on nano-sized extracellular vesicles, known as exosomes, to answer pivotal problems such as those mentioned above. Exosomes refer to a class of vesicles produced via the endocytic pathway and ranging in size from 30-140 nm in diameter. As a new piece to the puzzle of cancer, exosomes represent an important aspect of biological signaling between cells and as a means of novel biomarker identification strategies^4^. This is directly linked to the biofunctional cargo enriched in exosomes such as proteins, miRNAs, and lipids^5,6^.

To date there are only a handful of publications focusing on exosomes in the disease setting of mesothelioma. The initial steps towards this area were to analyze the proteomic make up of exosomes isolated from pleural effusions^7^ and separately by mesothelioma tumor cells^8^. A decade after these reports, it was shown that tumor-derived exosomes could be used in dendritic-cell (DC) based immunotherapeutic strategies against mesothelioma by treating tumor-bearing mice with DCs loaded with MM exosomes, showing that the exosomes imparted the mice with an immunological response against the MM thereby increasing survival rate^9^. Recently, Greening *et al*. added to the field that MM exosome signature reflects oncogenic cargo^10^. MicroRNAs (miRNAs) have been implicated in oncogenesis and could be exploited as potential therapeutic targets^11^.There have been multiple of investigations into the miRNAs involved in mesothelioma, particularly by Glen Reid’s group who has summarized a large swath of such knowledge and reported miRNA levels in MM tumor cells and tissues. Of note, the study indicated very low expression levels of tumor suppressor miRNAs in MM such as miR-16-5p, miR-15, miR-31, and let-7a, to name a few^12^. In another study by Oliveto *et al*.^13^ miR-24-3p has been identified as an oncomir that is pro-migratory in MM. Furthermore, diagnostic value of miRNAs in asbestos exposure and MM has also been proposed^14^ Notably, as per our knowledge only one current research article looks at the miRNA signature associated with circulating extracellular vesicle (EV) miRNAs in MM patients, and found that miR-103a-3p and miR-30e-3p were discriminatory for MM from asbestos-exposed patients with no cancer^15^.

Recently, our group has shown differential abundance of exosome proteomic signatures in mouse-serum after asbestos exposure^16^, and suggested a novel mechanism by which MM may develop by exosomes traveling from asbestos exposed cells to mesothelial cells thereby modifying the mesothelial cells’ gene expression patterns^17^.

Our present study is the first to present a quarry into the exosomal miRNAs of MM along with findings implicating new avenues of potential biomarkers and therapeutic options. Here, we investigate the signature miRNAs in MM tumor cell exosomes, and formulated a hypothesis that MM tumor cells preferentially secrete the tumor suppressor miR-16-5p via exosomes. Furthermore, we demonstrated that by inhibiting exosome secretion or force-feeding cancer exosomes back to MM cells can rebuild miR-16-5p levels in the cancer cells resulting in significant killing of cancer cells. Our findings may lead to potential therapeutic strategies for MM in future.

## Results

### Exosome isolation and characterization from MM cells

The isolation of exosome samples was characterized by transmission electron microscopy (TEM), Western blot analysis for exosomal marker CD81, and nanoparticle tracking analysis (NTA) (Supplementary Fig. 1). The vesicle isolates are predominantly in the size range of 30-140 nm in diameter as seen in TEM and NTA, and are enriched in CD81. TEM also indicates the well-described “cup-shape” morphology of the exosomes, and none of the exosomal samples showed a signal for calnexin, suggesting no presence of endoplasmic reticulum contaminants.

### Mesothelioma cancer cells secrete high levels of miR-16-5p in exosomes

**Figure 1:**
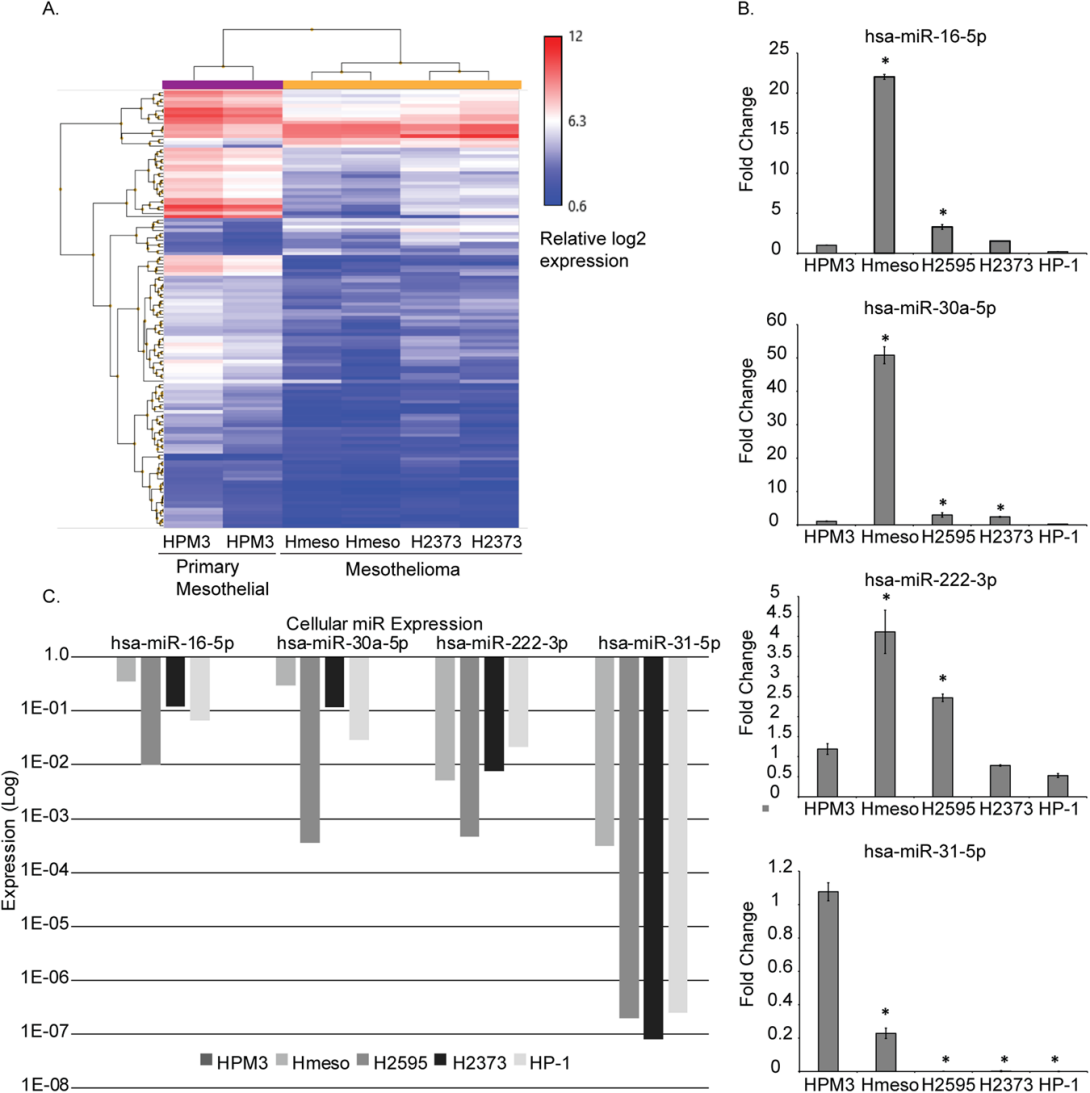
miRNA Array and validation of exosomal miRNAs from mesothelial and mesothelioma cells. (**A**) GeneChip miRNA 4.0 array heat map of miRNA abundances from human mesothelioma cell (Hmeso, H2373) exosomes as compared to normal primary human mesothelial cell (HPM3) exosomes. (**B**) qPCR validation of exosomal miR-16-5p, miR-30a-5p, miR-222-3p, and miR-31-5p in 4 MM cell lines compared to HPM3 cells. (**C**) Cellular miRNA expression of validated exosomal miRNAs in MM cell lines as compared to HPM3 cells by relative log decrease in expression, with control HPM3 expression levels set to 1.0. Number of replicates are 2 (n = 2), data is presented as mean ± SEM, and *p ≤ 0.05, by 1-way ANOVA as compared to HPM3 cells.

miRNA microarray profiling was conducted on isolated exosomal RNA from MM cell lines and primary mesothelial cells to compare non-cancer versus cancer signature (Fig. 1A). There were a total of 20 exosomal miRNAs upregulated and 110 downregulated in expression from MM tumor cell exosomes as compared to exosomes from primary mesothelial cells, with a > 2-fold cut-off for both parameters. All 130 miRNAs with > 2-fold differential expression are significant with ANOVA p-value < 0.05 (Supplementary Table 1 supplementary data).

We chose to validate the exosomal miRNA expression levels of 3 upregulated (miR-16-5p, miR-222-3p, miR-30a-5p) and one down regulated (miR-31-5p) miRNA by qRTPCR (Fig. 1B), all of which have been implicated in MM biology. Additionally, we validated exosomal miRNA by qRTPCR with 2 extra MM cell lines, H2595 (epithelioid subtype) and HP-1 (biphasic), along with the originally tested Hmeso and H2373, and show (Fig. 1B) that miR-16-5p, miR-222-3p, and miR-30a-5p are upregulated in exosomes from both epithelioid subtypes, Hmeso and H2595. The sarcomatoid H2373 showed upregulation only in miR-16-5p and miR-30a-5p, however, no upregulation was observed in the biphasic HP-1. miR-31a-5p was significantly decreased in all MM cancer exosomes.

We also performed qRTPCR to indicate the intracellular levels of each miRNA analyzed to show appropriate comparisons of producer cell quantities versus the amount secreted in exosomes, and found that all were significantly under-expressed as compared to the primary mesothelial cells HPM3 (Fig. 1C).

### Inhibited exosome secretion of MM cells attenuated tumorigenic properties

**Figure 2:**
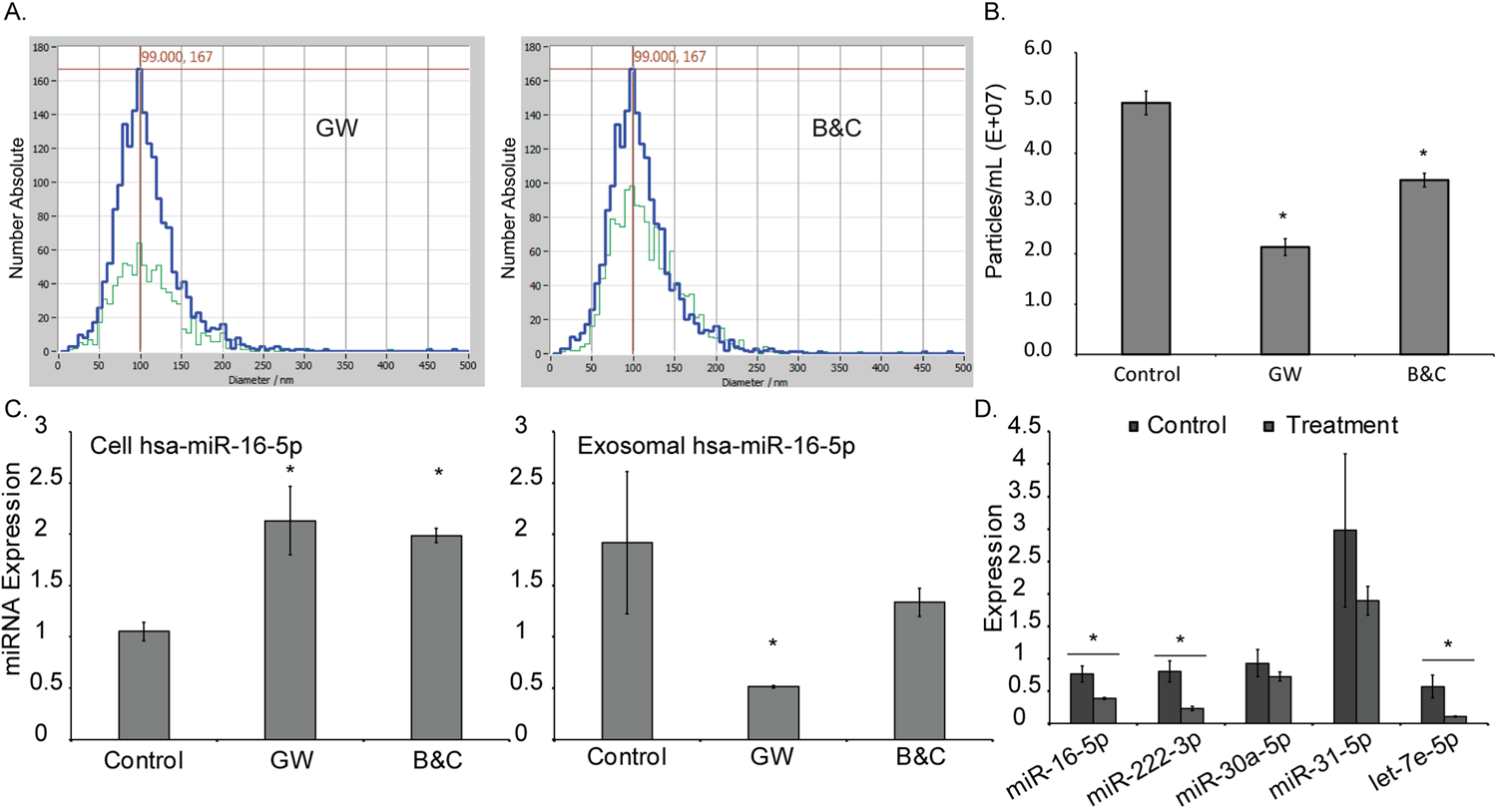
Inhibition of exosome secretion from Hmeso MM cancer cells attenuates exosomal miRNA secretion. (**A**) Nanoparticle tracking analysis plots of control (blue) exosomes overlaid with either GW4869 (GW) treated cell exosomes or Bisindolylmaleimide-I with Chloramidine (**B**,**C**) treated cell exosomes (green). (**B**) Particles/mL of control exosomes compared to both exosome inhibitor treatment groups, n = 3/group. (**C**) miR-16 expression in cells and exosomes after exosome secretion inhibition, n = 3. (**D**) qPCR validation of other miRNAs being reduced in MM exosomes after exosome inhibition by GW. All miRNA qPCR data is normalized to synthetic spike-in control cel-miR-39-3p, which was added to all exosome or cell isolates prior to RNA isolation. N = 2, mean ± SEM, and *p ≤ 0.05, by 1-way ANOVA or two-tailed Student’s t-test as compared to vehicle treated controls.

Treatment of Hmeso MM cells with small molecule inhibitors GW4869 (GW, 40 µM) or combination of Bisindolylmaleimide-I (10 µM) with Chloramidine (50 µM) (B&C), for 72 hours, resulted in significant reductions in exosome secretion from both treatment groups (Fig. 2A,B) as measured by NTA. Subsequent analysis of miR-16-5p provided with confirmation that inhibition of exosome secretion leads to concomitant reduction in secreted miR-16-5p in addition to increased levels of intracellular miR-16-5p within MM cancer cells (Fig. 2C). This result suggests that both inhibitors function differently in their capacity to effect exosome secretion and miR-16 secretion, and that neither inhibitor regulates levels of miR-16. If the inhibitors regulated miR-16 levels directly, we would expect to have seen intracellular levels of the miRNA altering in the same direction as observed in the exosomes; instead, they are increased in the cell and decreased in the exosomes. For further validation, we measured 4 other exosomally secreted miRNAs that were initially identified by microarray, and found that exosome inhibition also leads to reduced levels of exosomal miR-222-3p, miR-30a-5p, miR31-5p, and let-7e-5p (Fig. 2D), when measured from direct exosome preparations.

**Figure 3:**
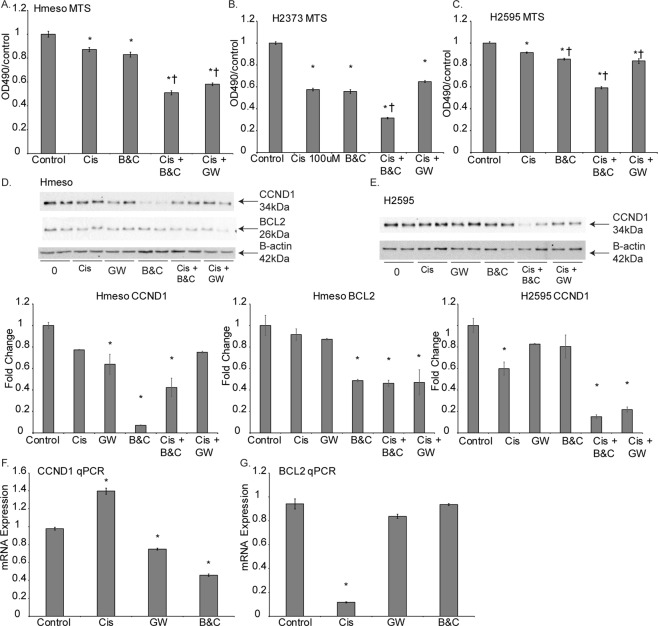
Inhibition of exosome secretion from MM cancer cells reduces proliferation and cellular abundance of oncogenic proteins targeted by miR-16. (**A**) MTS proliferation assay on Hmeso cells after cisplatin treatment, exosome inhibition, or combination of both treatments after 72 hr, n = 6. (**B**) and (**C**) MTS proliferation assay on H2595 and H2373 cells, respectively, after cisplatin treatment, exosome inhibition, or combination of both treatments after 72 hr, n = 6 (**D**) Immunoblot of Hmeso cellular proteins and miR-16 targets CCND1 and BCL2, normalized to β-actin. n = 2. (**E**) Immunoblot of H2595 cellular protein and miR-16 target CCND1, normalized to β-actin content. n = 2. qPCR of miR-16-5p oncogenes (**F**) CCND1 and (**G**) BCL2 after exosome inhibition, as normalized to HPRT endogenous control. *p ≤ 0.05 as compared to control, ^†^p ≤ 0.05 as compared to cisplatin treated group by 1-way ANOVA.

We further measured the viability of MM tumor cells (Hmeso, H2595, and H2373) using the MTS colorimetric assay following 72 hr treatment with exosome secretion inhibitors with and without cisplatin. As shown in Fig. 3A–C, B&C mediated exosome inhibition caused significant cell death by itself whereas GW did not lead to significant reduction in cell viability (data not shown). Both exosome secretion inhibitors led to significant reductions in cell numbers when combined with a low dose of cisplatin (Fig. 3A–C). Furthermore, Annexin-V expression, assayed by flow cytometry, was slightly elevated in all treatment groups compared to control and cisplatin alone, indicating that apoptosis alone does not explain the decrease in cell viability observed upon inhibition of exosome secretion (data not shown).

To elucidate further both the mechanism of reduced number of cells and the effects of miR-16-5p replenishment, we conducted Western blot analysis of miR-16-5p target proteins CCND1 and BCL2 in exosome secretion inhibited MM cells. Protein levels of both CCND1 and BCL2 decreased in exosome secretion inhibited Hmeso cells (Fig. 3D), and CCND1 was decreased in H2595 cells (Fig. 3E). BCL2 could not be detected for H2595 cells. At the mRNA level, we showed that exosome inhibition does not affect expression of CCND1 (Fig. 3G), although cisplatin does, and that BCL2 levels are significantly reduced upon exosome inhibition and increased with cisplatin (Fig. 3G).

The effect of exosome secretion inhibition with and without cisplatin also led to significant reductions in MM cell growth in 3-D spheroid models in both Hmeso (Supplementary Fig. 2 A-C) and H2373 cells (Supplementary Fig. 1D–F) as measured by size and MTS assay. Additionally, the migratory and invasive capacity for both Hmeso and H2373 cells was significantly reduced upon inhibition of exosome secretion (Supplementary Fig. 3).

### Force-feeding MM cancer exosomes back to MM producer cells attenuates tumorigenesis

**Figure 4:**
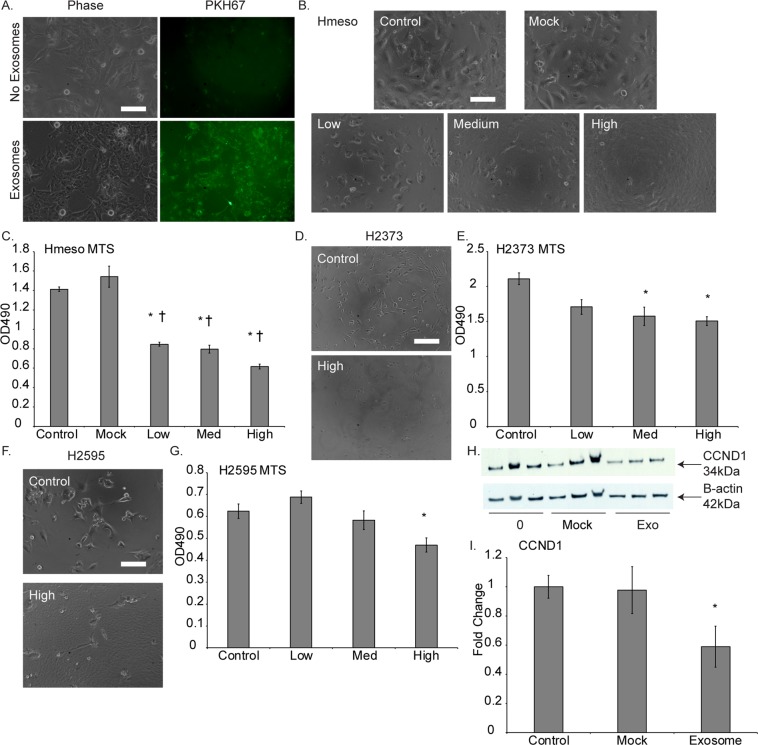
Force-feeding MM cancer cell exosomes back to MM cells leads to cancer cell death. (**A**) PKH67 labeled exosomes from Hmeso cells added to Hmeso cells show uptake/interaction of exosomes with target cancer cells. (**B**) Phase contrast images of Hmeso cells after addition of three different concentrations of Hmeso exosomes (concentrations based on volume of cell media collected from) or Mock (ExoQuick-TC), and (**C**) MTS proliferation assay of force feeding MM cancer exosomes, n = 6. Similar experiments with H2373 cells by (**D**) phase imaging and (**E**) MTS proliferation assay, as well as with H2595 MM cells by (**F**) phase imaging and (**G**) MTS proliferation assay, n = 6. Force feeding Hmeso exosomes to Hmeso MM cells (**H**) significantly reduced protein levels of CCND1 as analyzed by Western blot analysis. Western blot images shown are representative images and quantitation graphs of (**I**) CCND1 are combined quantitation of 3 repeated experiments. N = 3, Phase contrast images were taken with 40× objective lens, scale bar = 100 µm, mean ± SEM, *p ≤ 0.05 as compared to control and ^†^ p ≤ 0.05 as compared to Mock by 1-way ANOVA.

To validate our findings of retention of exosomes and decreased tumor characteristics, we ventured to assess if force-feeding the MM cancer exosomes back to the producer MM cells had a similar effect of reduced tumorigenesis via delivering back their secreted miR-16-5p. As a first step, we validated that MM exosomes interacted with, and were taken up by their own cells by addition of PKH67-labeled exosomes (Fig. 4A). All cell lines and their exosomes produced the same results of interaction and staining.

Next, we isolated exosomes from conditioned media after 72 hours in culture for MM exosomes to accumulate. The pelleted exosomes were suspended in 0.5% FBS (exosome-free) media at three different concentrations: low, medium, and high, based on the amount of media from which the exosomes were pelleted, 5 mL, 10 mL, and 20 mL, respectively. Suspended exosome pellets were added to 3,000 MM cells/well in 96-well plates and incubated overnight. The following day, cells were imaged and assayed for viability by MTS. The MM cells force-fed with their own concentrated exosomes endured significant amounts of cell death as observed by phase-contrast imaging and MTS assay (Fig. 4B-G). This was the case for both epithelioid MM subtypes (Hmeso and H2595) and the sarcomatoid subtype (H2373). As an additional control, we added a “mock” group of cells that were exposed to a mixture of exosome-free suspension media and ExoQuick-TC precipitation media after spinning alongside our true exosome isolates to verify that the ExoQuick-TC reagent was not having any effect on the cells. We found that the reagent/media mixture had no significant effect on target cells (Fig. 4B,C). Furthermore, we also confirmed that miR-16-5p target protein CCND1 was reduced in abundance (Fig. 4H,I).

To check for the selectivity of exosome force feeding response, we conducted force-feeding of cancer exosomes to non-cancer mesothelial cells (LP9) and also of non-cancer LP9 exosomes to themselves and to the Hmeso MM cancer cells. We found that MM cancer exosomes only killed the MM cancer cells from which they were produced and had no effect on LP9 mesothelial cells. LP9 exosomes had no effect on the proliferation of their producer cells and had a somewhat promotive growth effect on Hmeso cancer cells as analyzed by MTS proliferation assay (Supplementary Fig. 4).

### miR-16-5p overexpression in MM tumor cells inhibits MM tumorigenesis

**Figure 5:**
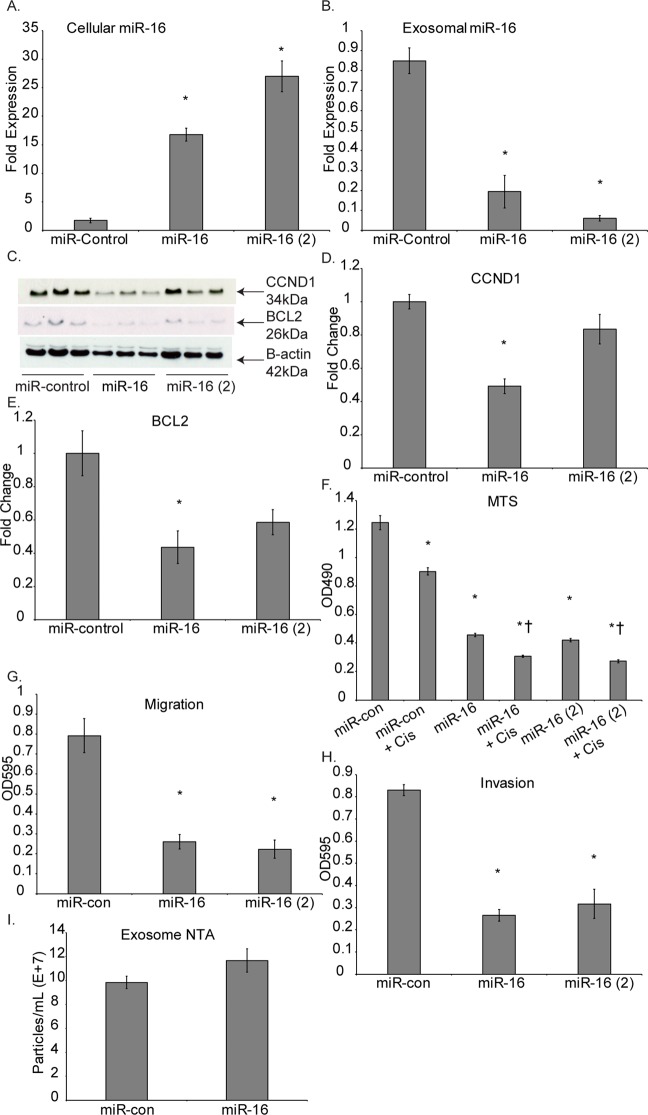
miR-16 overexpression in Hmeso cells leads to decreased MM cancer cell proliferation and protein abundance of CCND1 and BCL2. (**A**) Cellular levels of miR-16 increased after transfection, whereas, contradictorily, (**B**) miR-16 levels in exosomes significantly decreased upon transfection of miR-16 into Hmeso cells. n = 3. The target proteins of miR-16 (**C**) (immunoblot), (**D**) CCND1 and (**E**) BCL2 were reduced after miR-16 transfection. Transfection of miR-16 also resulted in (**F**) significantly reduced proliferation of MM cells by MTS, and increased cell death by cisplatin (n = 6), along with attenuated capabilities of (**G**) migration (n = 3) and (**H**) invasion (n = 3). (**I**) miR-16 transfection had no effect on secreted exosome numbers as assessed by NTA. All miRNA qPCR data is normalized to synthetic spike-in control cel-miR-39-3p, which was added to all exosome or cell isolates prior to RNA isolation. Mean + SEM, *p ≤ 0.05 as compared to control and ^†^p ≤ 0.05 as compared to cisplatin by 1-way ANOVA.

To ensure that the above effects seen with exosome secretion inhibition and force-feeding cancer exosomes was in fact due, at least in part, to miR-16-5p levels within the cancer cells we employed direct transfection of miR-16-5p mimics to Hmeso cells. Initial analysis determined that as expected miR-16-5p transfection led to significantly higher levels of intracellular miR-16-5p (Fig. 5A). We observed decreased levels of miR-16-5p in exosomes from transfected cells (Fig. 5B).

Further, overexpression of miR-16-5p within the cells resulted in significant reduction in miR-16-5p target proteins CCND1 and BCL2 as analyzed by Western blot (Fig. 5C–E). This result also coincided with significantly reduced viable Hmeso cells after miR-16 transfection alone as well as with cisplatin (Fig. 5F). To establish that transfection was not specific to only one particular set of miR-16-5p mimics, we transfected cancer cells with two separate miR-16-5p mimics from different lots and observed comparable results. The migratory and invasive capacity of Hmeso cells was also significantly abrogated upon miR-16-5p transfection (Fig. 5G,H). The observation of decreased levels of miR-16-5p in exosomes from transfected cells, suggests two possibilities; either secretion of exosomes is inhibited in response to transfection or loading of miR-16-5p into exosomes is decreased. Therefore, we conducted NTA from transfected experiments and found that the cells with over-expressed miR-16 upon transfection had no significant change in exosome secretion compared to control cells (Fig. 5I).

### HuR is possibly involved in loading miR-16 into exosomes of MM cells

Based on previous work done by other groups, it has been decisively implicated that the RNA binding protein HuR interacts with miR-16-5p^18^. Therefore, a further investigation into exosomal miR-16-5p was to uncover if the RNA binding protein HuR was involved in loading the miRNA into the vesicles. Immunostaining indicated that HuR was indeed present in the cytoplasm and nucleus of Hmeso MM cells (data not shown). Western blot analysis showed that no measurable amount of HuR was present in MM exosomes (data not shown).

**Figure 6:**
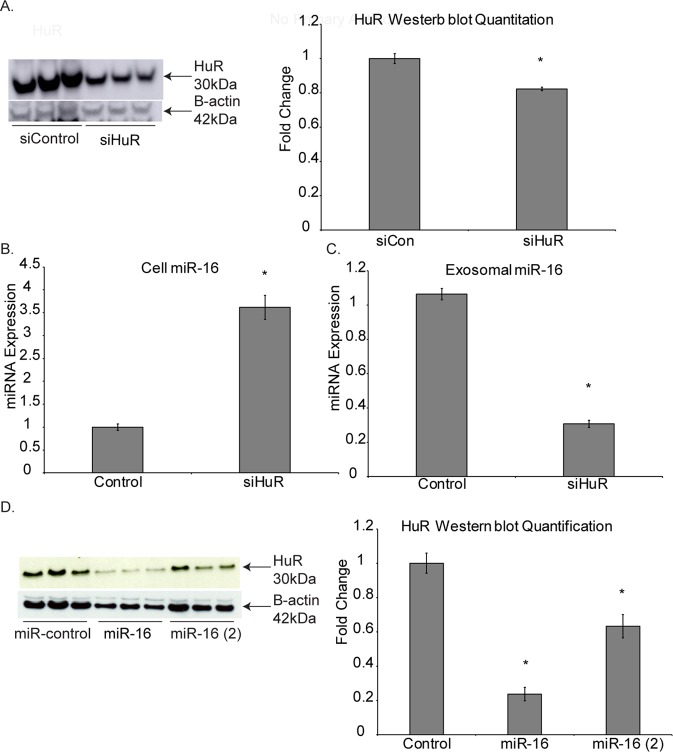
HuR is involved in the exosomal secretion of miR-16 from MM cells. (**A**) Transfection of siHuR significantly reduced the protein abundance of HuR as analyzed by Western blot analysis (**B**), and led to significantly increased cellular expression of miR-16 and (**C**) significantly decreased expression of miR-16 in exosomes. (**D**) Transfection of miR-16 mimic similarly induced significant reductions in HuR protein levels as analyzed by Western blot analysis (using the same immunoblot as Fig. 5, therefore B-actin is the same). n = 3. mean + SEM, *p ≤ 0.05 as compared to control by 1-way ANOVA.

Transfection of siHuR to Hmeso MM cells resulted in significant reduction in HuR protein levels (Fig. 6A). Additionally, HuR inhibition caused significantly higher levels of intracellular miR-16-5p and significantly lower levels of exosomal miR-16-5p (Fig. 6B,C) suggesting a possible role of HuR in exosomal miRNA chemistry. Interestingly, it has been shown that miR-16 may target the expression of HuR^19^, and our data also provides the same evidence by Western blot analysis of Hmeso cells transfected with miR-16-5p (Fig. 6D).

## Discussion

Malignant mesothelioma is a cancer of mesothelial cells caused by asbestos exposure, with dismal prognosis, and virtually no effective methods of early diagnosis or successful therapeutic approaches. Our hypothesis was to attack these knowledge gaps by embarking to uncover potential miRNA biomarkers and therapeutic targets with the focus on MM tumor exosomes. To date, exosomes have been a trending theme in cancer research and there have been wonderful advancements in understanding cancer through the lens of tumor exosomal miRNAs as possible biomarkers and therapies in many cancers^20–22^, however, not much is known about their role(s) in MM.

Our initial findings were surprising in that we observed MM tumor cells secreting significantly higher levels of tumor suppressor miRNAs, particularly miR-16-5p, compared to non-cancer mesothelial cells in exosomes. Previous studies of MM have well-characterized miR-16-5p as both a tumor suppressor by targeting oncogenes BCL2 and CCND1^23–25^, and as a remarkably under-expressed miRNA in MM tumor cells and tissues^12,26^. Moreover, miR-16-5p has been reported as a potential therapeutic molecule by restoring its expression in MM^27,28^. Based on these previously reported pieces of evidence on the role of miR-16-5p in MM, and our new findings of presence of miR-16-5p in exosomes led us to the innovative hypothesis that MM tumor cells preferentially secrete miR-16-5p, among other tumor suppressors, via exosomes to rid their cancer-killing effects. Similar results have been stated previously in ovarian cancer that may behave similarly in secreting high levels of tumor suppressor miRNAs^29^.

The other over-expressed tumor associated exosomal miRNAs also have interesting biological relevance in MM: miR-222-3p is downregulated in MM and is a negative regulator of CDK1 (p27) and is a PTEN suppressor^30^; miR-30a-5p (along with miR-222-3p and miR-31-5p), in the same family as miR-30e, is associated with good prognosis when in higher abundance in MM tumors^31^; and miR-320 family members are suggested as potential biomarkers for malignant pleural mesothelioma^32^.

This set of miRNAs along with miR-16-5p were under-expressed in the MM cells from which they were being secreted. Our findings are supported by literature that MM cells and tumors have low levels of tumor suppressor miRNAs including miR-16-5p^12^ as compared to mesothelial cells. This leads us to the possibility that miR-16-5p is not simply randomly packaged into cancer exosomes due to inherently high abundance in the exosome producer cells, but that it must be systematically sequestered into exosomes by a biologically preferential loading process. The low miR-16-5p in MM tumor cells observed by us and others could be attributed to its increased secretion via exosomes, as opposed to common loss of tumor suppressors via mutation and deletion.

Because of miR-16-5p’s tumor suppressive effect and our hypothesis of its preferential secretion from MM tumor cells by exosomes, we aimed to inhibit MM cell tumorigenesis by inhibiting exosome secretion. The idea being that if exosome secretion were significantly down-regulated, that miR-16-5p stores would rebuild in the tumor cells and lead to oncogenic targeting and subsequent cell death.

Based on successful outcomes in the literature we chose to use two separate exosome secretion inhibitory approaches. The neutral sphingolmyelinase-2 inhibitor GW4869, which blocks ceramide production that is needed to bud exosomes inward at the endosomal surface, has been indicated as a useful avenue for blocking exosome secretion as well as increasing the efficacy/reducing chemotherapeutic drug resistance to cisplatin^29,33,34^. Further, a combination of two small molecules bisindolylmaleimide-I and chloramidine have also been shown to reduce exosome secretion from cells and increase chemotherapeutic retention^35^.

Using GW or B&C treatment of Hmeso cells, led directly to significant reductions in exosomal miR-16-5p along with other miRNA secretions and significantly increased stores of cell cytoplasmic miR-16-5p. The inhibition of exosome release coupled with miR-16-5p retention in cells provided direct evidence that the miRNAs we were investigating were indeed being released in exosomes, and that we could prevent their preferential release and achieve the goal of miR-16-5p tumor suppressor retention. Also, we see that both inhibitors used, did not appear to negatively regulate the expression of miR-16-5p in the cells, as seen by the increased cellular miR-16-5p, so we can conclude that the drugs work by inhibiting exosomal miR-16-5p secretion and not the levels within the cell. Further confirmation of miR-16-5p retention was assessed by significantly reduced protein abundance of the miR-16-5p targets CCND1 and BCL2 and substantial inhibition in tumorigenesis as measured by loss of cell number, 3D tumor spheroid growth, transformation/colony growth, migratory and invasive capacity of MM tumor cells.

Additionally, we indicate that exosome secretion may play an important role in chemotherapeutic resistance of MM to cisplatin, given that exosome inhibition leads to increased cisplatin-induced cell death in our *in vitro* studies. In support to our findings, it has been reported before that chemotherapeutic drugs can be lost via exosomes^36,37^.

The logical next step for our studies was to show if we can feed back these exosomes to tumor cells and see the similar effects on tumorigenesis and confirm the findings of exosome secretion inhibition. All studies to date on the effect of tumor exosomes has indicated that they are pro-tumorigenic by multiple means (immunosuppression, drug resistance, enhanced tumor growth/proliferation, metastasis, angiogenesis, mesenchymal/fibroblastic transitions etc.)^22,38–44^. However, our hypothesis was different because of our intriguing findings that MM tumor exosomes have high volumes of tumor suppressor miRNAs, especially miR-16-5p, and may have a tumor killing effect.

Remarkably, addition of MM exosomes back to the producer MM cells, led to incredible levels of cell death in a dose dependent manner as compared to controls. Further, upon force-feeding of MM cancer exosomes to cancer cells, we see significant decreases in miR-16-5p target oncogenic protein CCND1. This is a vital result in that it shows that we can demonstrate the same result as exosome inhibition but in a separate route. Importantly, MM cancer exosome force-feeding, even more notably, was only seen to kill MM cancer cells and had no effect on normal mesothelial cells. Also, normal mesothelial cell exosomes had no effect on cancer cells or on themselves when force-fed back.

These findings are the first of their kind, to show that not only can a certain cancer’s exosomes lead to the death of their producer cells, but they do so in a specific manner that does not affect non-cancer cells. The implications of this are exciting in that they may suggest a very new therapeutic option in MM by targeting tumor cells with their own exosomes. This is especially noteworthy given that the effect of MM exosomes on the cancer cells is implicated in the effect of the tumor suppressor miR-16-5p that is functioning in the exosome inhibition experiments.

We do understand that we are drawing our discussion of force-feeding exosomes based only on one component of what is being redelivered to the tumor cells. In reality, the exosomes will be delivering back a vast array of molecules in combination to the miR-16-5p and other tumor suppressors, and those effects should be considered. Essentially, we know that miR-16-5p plays a role in this complicated biology, but there is likely a lot of interplay with everything else within the MM tumor exosomes that needs to be studied.

Moreover, we recapitulated the same findings from exosome inhibition and force-feeding via direct transfection of miR-16-5p, providing firm evidence that not only is exosome secretion a pro-tumorigenic mechanism of MM by preferential secretion of miR-16-5p but that exosomal miR-16-5p is of potential therapeutic importance as reported before^12,28,45^. As a counterintuitive piece of evidence, we did see that although miR-16-5p levels increased intracellularly in MM cancer cells after transfection of miR-16-5p mimic, the levels in their exosomes significantly plummeted. This may suggest that miR-16-5p levels may also play a role in the loading or secretion of exosomes with miR-16-5p within. As NTA analysis showed no significant differences in the number of particles in response to miR-16-5p transfection, there is a further possibility of miR-16-5p exhibiting an effect on packaging/loading system of exosomes.

Here we also intended to unlock the potential mechanism for miR-16 loading into exosomes within MM cancer cells. Based on previous reports of RNA binding protein HuR interaction with miR-16-5p^18^ and evident role in exosome secretion of other miRNAs^46^, we explored its role in our experimental settings. Significant reduction in HuR expression via siRNA lead to increased amount of miR-16-5p in cells, and significantly reduced the amount secreted in exosomes. This result is evidence that HuR is at least partly involved in the packaging of miR-16-5p into MM cancer exosomes.

Furthermore, previous research has also shown that miR-16 targets the expression of HuR^19^, we also provide this same evidence as measured by Western blot analysis, telling us something very interesting about the mechanistics of miR-16-5p exosomal loading and the interplay of HuR. Based on our results thus far, we have drawn together a proposed mechanism that may be involved where HuR promotes miR-16-5p loading into exosomes, meaning that siHuR knockdown of HuR protein levels leads to reduced exosomal miR-16-5p, and hence increased miR-16-5p in the cell. It is then implicated that miR-16-5p targets HuR expression at the protein level, meaning that high miR-16-5p levels lead to low HuR and therefore, reduced exosomal miR-16-5p, which is exactly what our data represents. Because miR-16-5p upregulation by transfection does not affect exosome secretion by particle number, we know that this effect is limited to the packaging process of exosome cargo. Along this logic, we theorize that MM cancer cells not only have the evolutionary advantage for uncontrolled cell growth because of low intracellular stores of miR-16-5p based on high exosomal removal, but that miR-16-5p itself negatively regulates its own packaging into exosomes by targeting HuR (Supplemental Fig. 5).

Taken together, our findings strongly indicate that miR-16-5p is preferentially secreted by MM tumor cells via exosomes *in vitro*, and by inhibiting exosome secretion, miR-16-5p levels increase thereby reducing oncogenic protein levels and lead to significant loss of tumorigenic capacity of the MM cancer cells. We also indicate that the mechanism of exosomal miR-16-5p secretion is at least somewhat regulated by HuR, and that there is a negative feedback loop involved in this packaging when miR-16-5p levels are increased within the cell.

Altogether, this novel research study provided unprecedented indications that MM tumor exosomes can be used to inhibit tumorigenesis, and this is related to the fact that the MM tumor cells preferentially secrete miR-16-5p through their exosomes to rid themselves of its tumor suppressor function. A recent study published by Guo *et al*.^47^ supports our concept that autologous tumor cell-derived particles (eg. exosomes) can be a promising therapeutic target for treating malignancies. As discussed above there are limitations to this study as exosomes contain lot more than just tumor suppressor miRNA. Our future endeavors include a series of *in vivo* experiments such as using exosome inhibition and exosome force-feeding in mice allografted with MM tumors to see if any effect can be seen in a whole organism.

## Materials and Methods

### Cell culture

Human pleural MM cell lines, H2373, H2595, and HP-1 were kindly contributed by Dr. Harvey Pass (New York University, New York, NY)^48^ and Hmeso cells were isolated by Reale *et al*.^49^. Human primary pleural mesothelial cells HPM3 and human immortalized peritoneal mesothelial LP9/TERT-1 (LP9) cells were purchased from Brigham and Women’s Hospital, Harvard University, Boston, MA.

All cell lines were cultured as previously reported^50^. Cell lines were validated by STR DNA fingerprinting using the Promega CELL ID System (Promega, Madison, WI)^50^.

Cisplatin was purchased from Alfa Aesar (Ward Hill, MA) and concentrations for the present study were selected based on previously published literature for MM cells^51^. GW4869, Cl-amidine (chloramidine), and bisindolylmaleimide-I were purchased from Cayman Chemical (Ann Arbor, MI) and used at concentrations based on published reports indicating successful inhibition of exosome release from cells^34,35^. DMSO in equal volume added to control wells as vehicle control.

### Immunostaining of MM cells for HuR

Hmeso cells were fixed in 4% PFA, blocked, washed, and incubated overnight at 4 °C with HuR antibody (Cell Signaling Technology, Danvers, MA) as previously described^50^. For a negative control, one slide was stained as described, excluding primary antibody. After further washing, cells were incubated with a fluorescently conjugated secondary antibody, AlexaFluor® 647 (Thermo Fisher, Grand Island, NY). Following nuclear staining with DAPI (Thermo Fisher), sections were imaged with a Nikon A1R-ER Confocal Microscope.

### Exosome isolation and characterization

#### Exosome isolation from cell culture medium

Exosomes were isolated using ExoQuick-TC precipitation reagent (System BioSciences, Palo Alto, CA, USA), as previously described^17^.

#### Transmission electron microscopy (TEM)

Double membrane structure of exosomes and size was confirmed using transmission electron microscope (JEOL 1400 TEM) as previously published^17^.

#### Nanoparticle tracking analysis

Exosomes number and size were further assessed by nanoparticle tracking analysis (NTA) using the ZetaView PMX 110 (Particle Metrix, Meerbusch, Germany) and Software ZetaView 8.02.31^17^.

#### Characterization of exosomes by Western blot analysis

Two aliquots of isolated exosomes from representative groups were characterized by immunoblot analysis for presence of exosomal marker CD81 (Sigma Aldrich) and also for absence of calnexin (Novus Biologicals, Littleton, CO, USA) to rule out contaminating ER vesicles^17^.

### MicroRNA isolation and microarray

Isolation of miRNA from exosome pellets was accomplished using Qiagen miRNeasy Micro Kit (Venlo, Netherlands) by adding QiaZol reagent directly to pellets and following the manufacturer’s protocol.

RNA quality from exosomes was assessed prior to microarray analysis using the Agilent 2100 Bioanalyzer (Agilent, Santa Clara, CA, USA), and subsequently the RNA was analyzed using using GeneChip™ miRNA 4.0 Array (Thermo Fisher Scientific, Waltham, MA, USA) performed on exosomal miRNA from HPM3, Hmeso and H2373 cells (n = 2). Data was analyzed using Transcriptome Analysis Console 4.0 (Thermo Fisher). Parameters were set to any gene that was expressed differently by both 2 fold and 1.5 fold up or down with an ANOVA p-value less than 0.05.

Validation of expression changes in selected miRNAs of interest was conducted by qRTPCR after cDNA synthesis from exosomal miRNA (normalized to 2uL exosome miRNA or 2 ng miRNA from cells) using TaqMan Advanced cDNA miRNA cDNA Synthesis Kit (Thermo Fisher, Waltham, MA) following the manufacturer’s protocol. We used TaqMan Assays on Demand primers and probes for human miRNAs miR-16-5p, miR-30a-5p, miR-222-3p, and miR-31-5p, and for internal control miRNA cel-miR-39-3p (Thermo Fisher) was used.

### Exosome secretion inhibition from cells

Exoxome secretion from cells was inhibited by using 2 different small molecule inhibitors described above.

### Immunoblot analysis

Cellular proteins of interest (miR-16-5p targets and HuR) were assessed by immunoblot analysis using antibodies specific to CCND1 and BCL-2 (Abcam) or HuR (Cell Signalling) as previously published^50^. Proteins selected for immunoblot analysis were of biological relevance as targets of miR-16-5p regulation.

### MTS assay

Cell viability was determined in various experiments by MTS Assay CellTiter 96 Aqueous One Solution Cell Proliferation Assay (Promega) as per the manufacturer’s recommendations^52^.

### *In Vitro* tumorigenic assays

Mesothelioma cells were treated with cisplatin/exosome inhibitors/transfected with miRNA mimics, and were assessed for various tumorigenic assays as described below.

#### 3-D model to grow mesothelioma spheroids

Mesothelioma cells were grown in a 3-D model using the Cultrex 3-D Spheroid Colorimetric Proliferation/Viability Assay from Trevigen, Inc. (Gaithersburg, MD). Mesothelioma cells were seeded at a density of 2,500/well following the manufacturer’s protocol. Six days later colorimetric analysis (MTT) was performed as stated in the manufacturer’s protocol^52^.

#### Migration assay

Migration of MM cells was assessed using 6-well Transwell polycarbonate filters (Corning Costar Corp., Corning, NY) with an 8-μm pore size as described previously

#### Invasion assay

Invasiveness of MM cells was assessed using 24-well Transwell polycarbonate filters (Corning Costar Corp., Corning, NY) with an 8-μm pore size with 1 mg/mL Matrigel coating gel on upper well as described previously^50^.

### siRNA and miRNA transfection

On-Target plus Non-Targeting small-interfering RNA (siRNA) (scrambled control) or On-Target plus SMARTpool human ELAV1 (HuR) siRNA (100 nmol/L, Dharmacon, Lafayette, CO) were transfected into 95% confluent cells using Lipofectamine RNAiMAX (Invitrogen), following the manufacturer’s protocol. The efficiency of HuR protein knockdown was determined by Western blot analysis after 48 hr. Two separate lots of siRNA were used in duplicate for each siHuR experiment.

MISSION miRNA mimic miR-16-5p and MISSION miRNA negative control were transfected into 95% confluent cells using Lipofectamine RNAiMAX (Invitrogen), following the manufacturers protocol. Two lots of miR-16 mimic were used in duplicate for each transfection experiment.

Success of transfection was verified by protein or RNA levels of transfected RNA.

### Exosome uptake by MM cells

Exosomes were labeled using PKH67 dye (Sigma Aldrich) according to the manufacturer’s protocol. Labeled exosomes were suspended in PBS and added to target cells and imaged on an Olympus IX70 inverted light microscope as described previously^17^.

### Exosome force feeding to MM cells

Exosomes were isolated from Hmeso MM cancer cells and equal volumes of exosome preparation rather than protein content from different groups were added to Hmeso cells. After 24 hr of exposure with exosomes, cells were imaged by phase contrast microscopy with 20× objective lens and subsequently analyzed by MTS assay or cell protein lysate was used for immunoblot.

### Statistical analysis

All experiments were performed in duplicate or triplicate and repeated at least twice. A one-way analysis of variance (ANOVA) followed by a Newman-Keuls procedure for adjustment of multiple pairwise comparisons or the student’s unpaired two-tailed t-test was applied to all data to establish the significance of observed differences between the various experimental groups. p ≤ 0.05 was considered significant. All statistical analyses were performed using the GraphPad Prism software program version 7.0 (GraphPad Software, La Jolla, CA).

## Supplementary information

Supplementary Figures

Microarray dataset

## Data Availability

All data generated or analyzed during this study are included in this published article (and its Supplementary Information files).

## References

[1] Mossman BT (2013). New insights into understanding the mechanisms, pathogenesis, and management of malignant mesotheliomas. The American journal of pathology.

[2] Rossini M (2018). New Perspectives on Diagnosis and Therapy of Malignant Pleural Mesothelioma. Frontiers in oncology.

[3] Yap TA, Aerts JG, Popat S, Fennell DA (2017). Novel insights into mesothelioma biology and implications for therapy. Nature reviews. Cancer.

[4] Munson P, Shukla A (2015). Exosomes: Potential in Cancer Diagnosis and Therapy. Medicines.

[5] Yu Dan-dan, Wu Ying, Shen Hong-yu, Lv Meng-meng, Chen Wei-xian, Zhang Xiao-hui, Zhong Shan-liang, Tang Jin-hai, Zhao Jian-hua (2015). Exosomes in development, metastasis and drug resistance of breast cancer. Cancer Science.

[6] Brinton LT, Sloane HS, Kester M, Kelly KA (2015). Formation and role of exosomes in cancer. Cellular and molecular life sciences: CMLS.

[7] Bard MP (2004). Proteomic analysis of exosomes isolated from human malignant pleural effusions. American journal of respiratory cell and molecular biology.

[8] Hegmans JP (2004). Proteomic analysis of exosomes secreted by human mesothelioma cells. The American journal of pathology.

[9] Mahaweni Niken M., Kaijen-Lambers Margaretha E.H., Dekkers Jacqueline, Aerts Joachim G.J.V., Hegmans Joost P.J.J. (2013). Tumour-derived exosomes as antigen delivery carriers in dendritic cell-based immunotherapy for malignant mesothelioma. Journal of Extracellular Vesicles.

[10] Greening DW (2016). Secreted primary human malignant mesothelioma exosome signature reflects oncogenic cargo. Scientific reports.

[11] Oliveto S, Mancino M, Manfrini N, Biffo S (2017). Role of microRNAs in translation regulation and cancer. World journal of biological chemistry.

[12] Reid G (2015). MicroRNAs in mesothelioma: from tumour suppressors and biomarkers to therapeutic targets. Journal of thoracic disease.

[13] Oliveto S (2018). A Polysome-Based microRNA Screen Identifies miR-24-3p as a Novel Promigratory miRNA in Mesothelioma. Cancer research.

[14] Micolucci L, Akhtar MM, Olivieri F, Rippo MR, Procopio AD (2016). Diagnostic value of microRNAs in asbestos exposure and malignant mesothelioma: systematic review and qualitative meta-analysis. Oncotarget.

[15] Cavalleri T (2017). Plasmatic extracellular vesicle microRNAs in malignant pleural mesothelioma and asbestos-exposed subjects suggest a 2-miRNA signature as potential biomarker of disease. PLoS ONE.

[16] Munson P, Lam YW, MacPherson M, Beuschel S, Shukla A (2018). Mouse serum exosomal proteomic signature in response to asbestos exposure. Journal of cellular biochemistry.

[17] Munson Phillip, Lam Ying-Wai, Dragon Julie, MacPherson Maximilian, Shukla Arti (2018). Exosomes from asbestos-exposed cells modulate gene expression in mesothelial cells. The FASEB Journal.

[18] Young LE, Moore AE, Sokol L, Meisner-Kober N, Dixon DA (2012). The mRNA stability factor HuR inhibits microRNA-16 targeting of COX-2. Molecular cancer research: MCR.

[19] Xu F (2010). Loss of Repression of HuR Translation by miR-16 May Be Responsible for the Elevation of HuR in Human Breast Carcinoma. Journal of cellular biochemistry.

[20] Rabinowits G, Gercel-Taylor C, Day JM, Taylor DD, Kloecker GH (2009). Exosomal microRNA: a diagnostic marker for lung cancer. Clinical lung cancer.

[21] Chevillet JR (2014). Quantitative and stoichiometric analysis of the microRNA content of exosomes. Proceedings of the National Academy of Sciences of the United States of America.

[22] Melo SA (2014). Cancer exosomes perform cell-independent microRNA biogenesis and promote tumorigenesis. Cancer cell.

[23] Cimmino A (2005). miR-15 and miR-16 induce apoptosis by targeting BCL2. Proceedings of the National Academy of Sciences of the United States of America.

[24] Wang DW, Wang YQ, Shu HS (2018). MiR-16 inhibits pituitary adenoma cell proliferation via the suppression of ERK/MAPK signal pathway. European review for medical and pharmacological sciences.

[25] Kao SC (2017). Tumor Suppressor microRNAs Contribute to the Regulation of PD-L1 Expression in Malignant Pleural Mesothelioma. Journal of thoracic oncology: official publication of the International Association for the Study of Lung Cancer.

[26] De Santi C (2017). Deregulation of miRNAs in malignant pleural mesothelioma is associated with prognosis and suggests an alteration of cell metabolism. Scientific reports.

[27] Reid G (2013). Restoring expression of miR-16: a novel approach to therapy for malignant pleural mesothelioma. Annals of oncology: official journal of the European Society for Medical Oncology.

[28] Viteri, S. & Rosell, R. An innovative mesothelioma treatment based on miR-16 mimic loaded EGFR targeted minicells (TargomiRs). *Translational Lung Cancer Research*, S1–S4 (2017). 10.21037/tlcr.2017.12.01 PMC5835633 29531894

[29] Kanlikilicer P (2016). Ubiquitous Release of Exosomal Tumor Suppressor miR-6126 from Ovarian Cancer Cells. Cancer research.

[30] Truini A (2014). Role of microRNAs in malignant mesothelioma. Cellular and Molecular Life Sciences.

[31] Birnie KA, Prêle CM, Thompson PJ, Badrian B, Mutsaers SE (2017). Targeting microRNA to improve diagnostic and therapeutic approaches for malignant mesothelioma. Oncotarget.

[32] Quinn L, Finn SP, Cuffe S, Gray SG, Non-coding RNA (2015). repertoires in malignant pleural mesothelioma. Lung cancer (Amsterdam, Netherlands).

[33] Kosaka N (2010). Secretory Mechanisms and Intercellular Transfer of MicroRNAs in Living Cells. J Biol Chem.

[34] Essandoh K (2015). Blockade of exosome generation with GW4869 dampens the sepsis-induced inflammation and cardiac dysfunction. Biochimica et biophysica acta.

[35] Kosgodage Uchini, Trindade Rita, Thompson Paul, Inal Jameel, Lange Sigrun (2017). Chloramidine/Bisindolylmaleimide-I-Mediated Inhibition of Exosome and Microvesicle Release and Enhanced Efficacy of Cancer Chemotherapy. International Journal of Molecular Sciences.

[36] Marleau AM, Chen C-S, Joyce JA, Tullis RH (2012). Exosome removal as a therapeutic adjuvant in cancer. Journal of translational medicine.

[37] Safaei R (2005). Abnormal lysosomal trafficking and enhanced exosomal export of cisplatin in drug-resistant human ovarian carcinoma cells. Molecular cancer therapeutics.

[38] Valenti R (2007). Tumor-released microvesicles as vehicles of immunosuppression. Cancer research.

[39] Iero M (2008). Tumour-released exosomes and their implications in cancer immunity. Cell death and differentiation.

[40] Webber J, Steadman R, Mason MD, Tabi Z, Clayton A (2010). Cancer exosomes trigger fibroblast to myofibroblast differentiation. Cancer research.

[41] Zhang HG, Grizzle WE (2011). Exosomes and cancer: a newly described pathway of immune suppression. Clinical cancer research: an official journal of the American Association for Cancer Research.

[42] Kosaka N (2014). Dark side of the exosome: the role of the exosome in cancer metastasis and targeting the exosome as a strategy for cancer therapy. Future oncology (London, England).

[43] Adorno-Cruz V (2015). Cancer stem cells: targeting the roots of cancer, seeds of metastasis, and sources of therapy resistance. Cancer research.

[44] Zhao L, Liu W, Xiao J, Cao B (2015). The role of exosomes and “exosomal shuttle microRNA” in tumorigenesis and drug resistance. Cancer letters.

[45] van Zandwijk N (2017). Safety and activity of microRNA-loaded minicells in patients with recurrent malignant pleural mesothelioma: a first-in-man, phase 1, open-label, dose-escalation study. The Lancet. Oncology.

[46] Mukherjee K (2016). Reversible HuR-microRNA binding controls extracellular export of miR-122 and augments stress response. EMBO Rep.

[47] Guo, M. *et al*. Autologous tumor cell-derived microparticle-based targeted chemotherapy in lung cancer patients with malignant pleural effusion. *Science translational medicine* **11**, 10.1126/scitranslmed.aat5690 (2019). 10.1126/scitranslmed.aat5690 30626714

[48] Pass HI (1995). Characteristics of nine newly derived mesothelioma cell lines. The Annals of thoracic surgery.

[49] Reale FR (1987). Characterization of a human malignant mesothelioma cell line (H-MESO-1): a biphasic solid and ascitic tumor model. Cancer research.

[50] Shukla A (2013). Extracellular signal-regulated kinase 5: a potential therapeutic target for malignant mesotheliomas. Clinical cancer research: an official journal of the American Association for Cancer Research.

[51] Hillegass JM (2011). Increased efficacy of doxorubicin delivered in multifunctional microparticles for mesothelioma therapy. International journal of cancer.

[52] Thompson JK (2018). Extracellular signal regulated kinase 5 and inflammasome in progression of mesothelioma. Oncotarget.

