# Supplementary material for: Exosomal miR-16-5p as a target for malignant mesothelioma
Source: Sci Rep. 2019 Aug 12;9:11688. doi: 10.1038/s41598-019-48133-0 (PMC6690928; doi:10.1038/s41598-019-48133-0)

Exosomal miR-16-5p as a target for malignant mesothelioma

Phillip B. Munson[1, 3], Elizabeth M. Hall[1], Nicholas H. Farina[2, 3], Harvey I. Pass[4], and Arti Shukla[1, 3]

[1]Department of Pathology and Laboratory Medicine, University of Vermont, College of Medicine, Burlington, VT 05405, USA

[2]Department of Biochemistry, University of Vermont, College of Medicine, Burlington, VT 05405, USA

[3]University of Vermont Cancer Center, University of Vermont, College of Medicine, Burlington, VT 05405, USA

[4]Department of Cardiothoracic Surgery, NYU Langone Medical Center, 530 First Avenue, 9V New York, New York 10016, USA

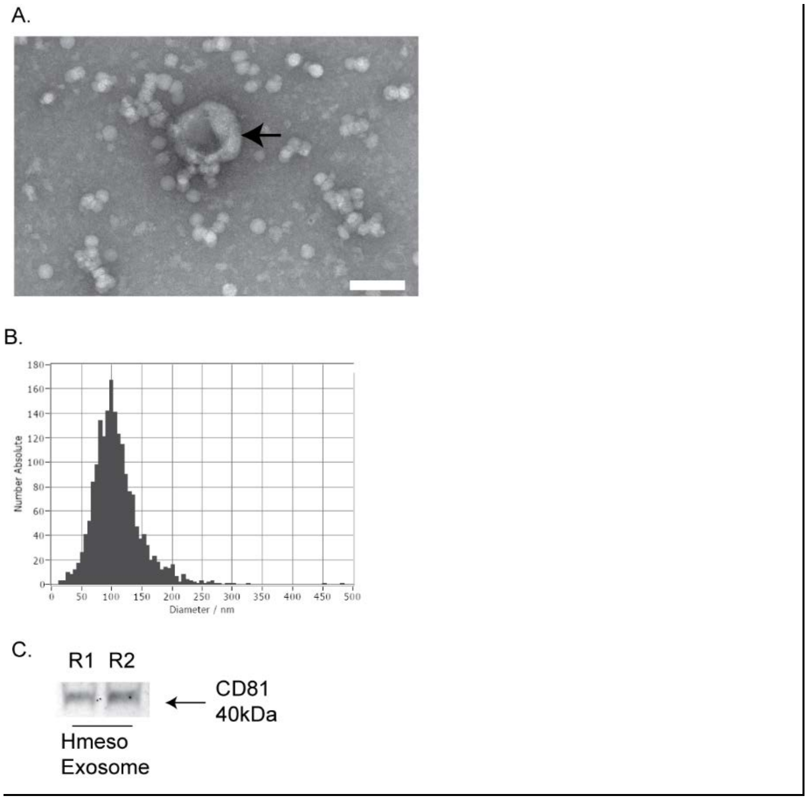

Supplementary Figure 1. Hmeso cell exosome characterization. A) TEM showing exosome membrane-bound structures and size range; indicated by arrow in middle of field. Scale bars, 100 nm. B) Nanoparticle Tracking Analysis (NTA) indicating exosome size distribution and concentration of particles. C) Western blot analysis for presence of exosome marker CD81 from two replicate (R1 and R2) exosome preparations from Hmeso conditioned cell culture supernatant, normalized by equal volume of exosomal input from equal cell number.

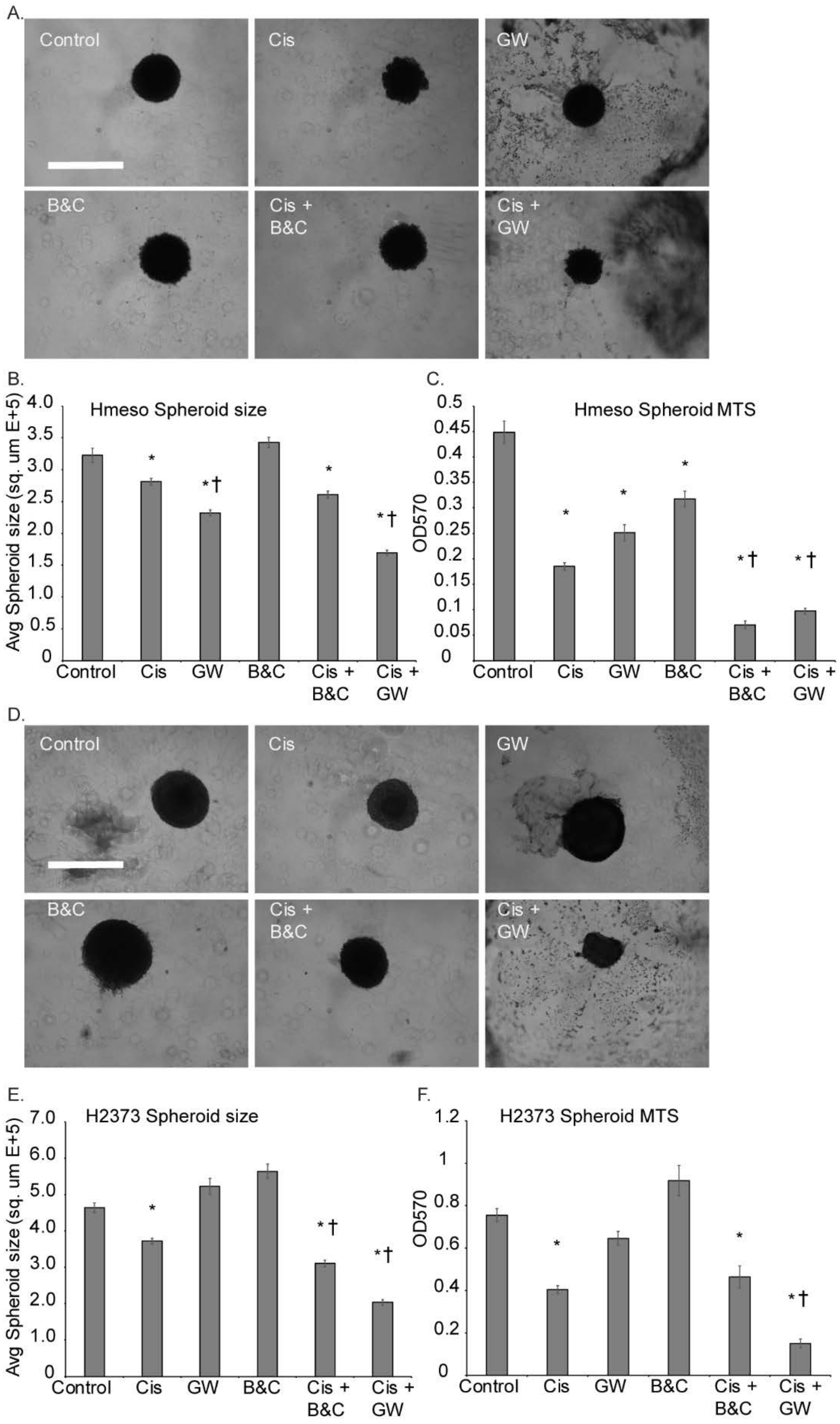

Supplementary Figure 2. Exosome secretion inhibition from MM cancer cells inhibits tumor 3D spheroid growth. A) Hmeso cells were grown as 3D spheroids as

described in materials and methods section and treated with cisplatin (Cis, 20µM), GW4869 (GW, 40µM), combination of Bisindolylmaleimide-I 10µM with Chloramidine 50µM (B&C), or combinations. Phase contrast images were taken after 6 days of growth with 20× objective lens, scale bar, 500µM B) 3D spheroid sizes were measured in ImageJ, and C) solubilized and measured for proliferation using MTS assay. D-F) Similar experiments with H2373 cells n=3, mean+ SEM, *p ≤ 0.05 as compared to vehicle control and † p ≤ 0.05 as compared to Cis alone by 1-way ANOVA

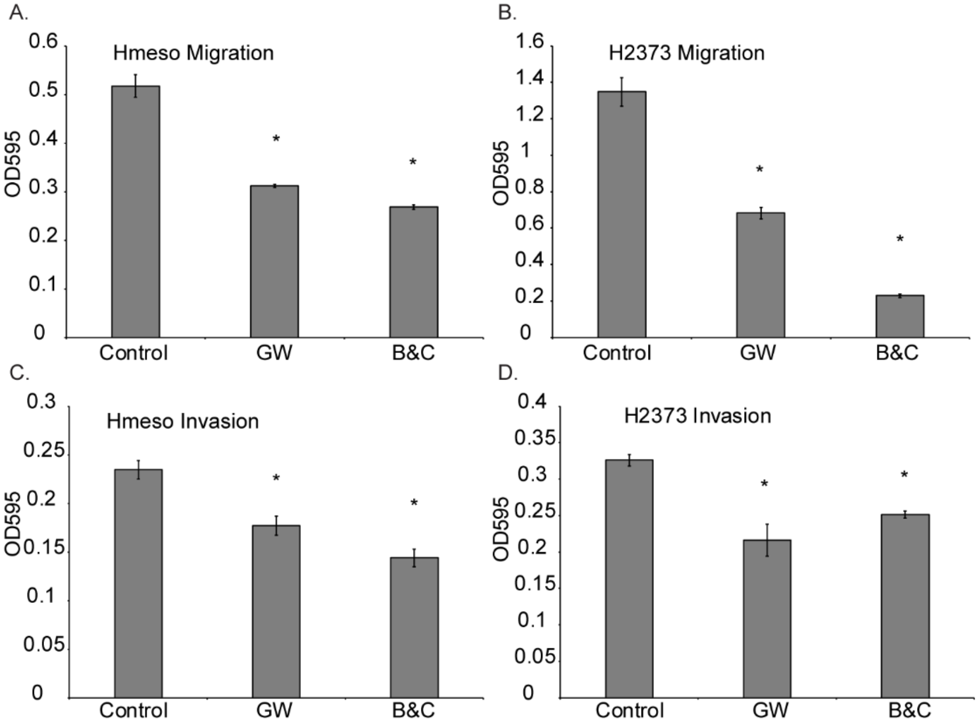

Supplementary Figure 3. Exosome secretion inhibition from MM cancer cells attenuated migration and invasion. MM cells were treated with exosome secretion inhibitors and then put on tranwells to assess migration /invasion after 72 h as described in the materials and method section. Inhibition of exosome secretion attenuated migration (A, B) and invasion (C, D) of Hmeso and H2373 MM cells. n=3, mean+ SEM, *p ≤ 0.05 as compared to vehicle control by 1-way ANOVA.

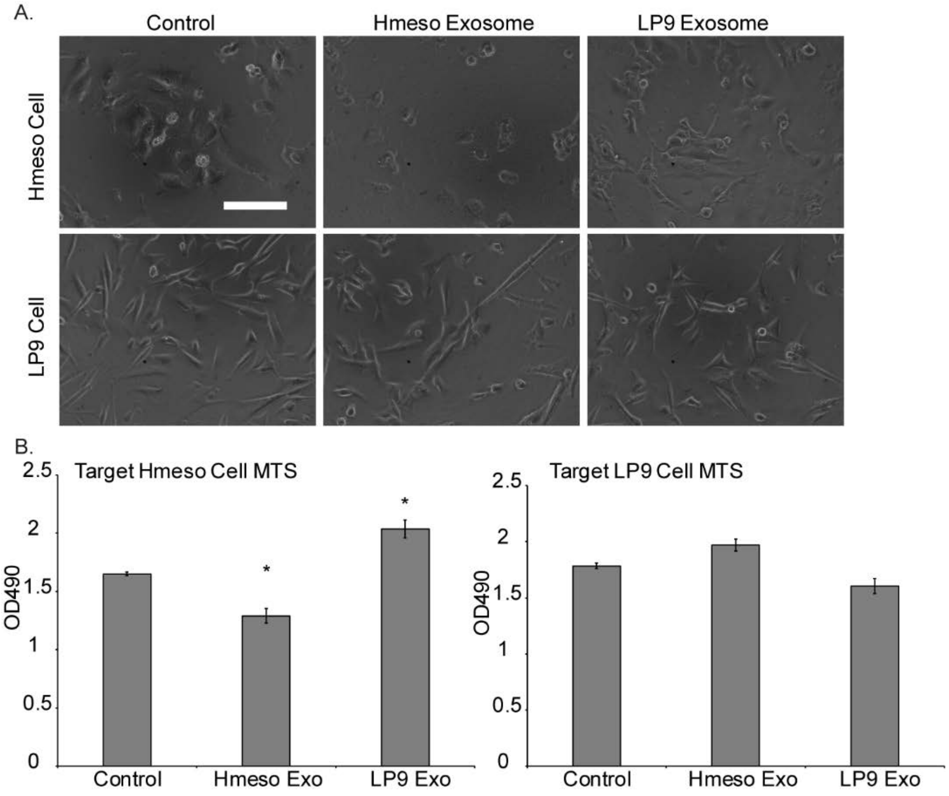

Supplementary Figure 4. Exosome force-feeding leads to cancer cell specific effects. A) Phase contrast images of control and force-fed mesothelial (LP9) or mesothelioma (Hmeso) cells in various combinations and B) MTS proliferation assay of force-feeding MM cancer exosomes and LP9 mesothelial cell exosomes to either target Hmeso MM cells or target LP9 cells. n=6, Phase contrast images were taken with 40× objective lens, scale bar = 100µm, mean± SEM, *p ≤ 0.05 as compared to control by 1-way ANOVA.

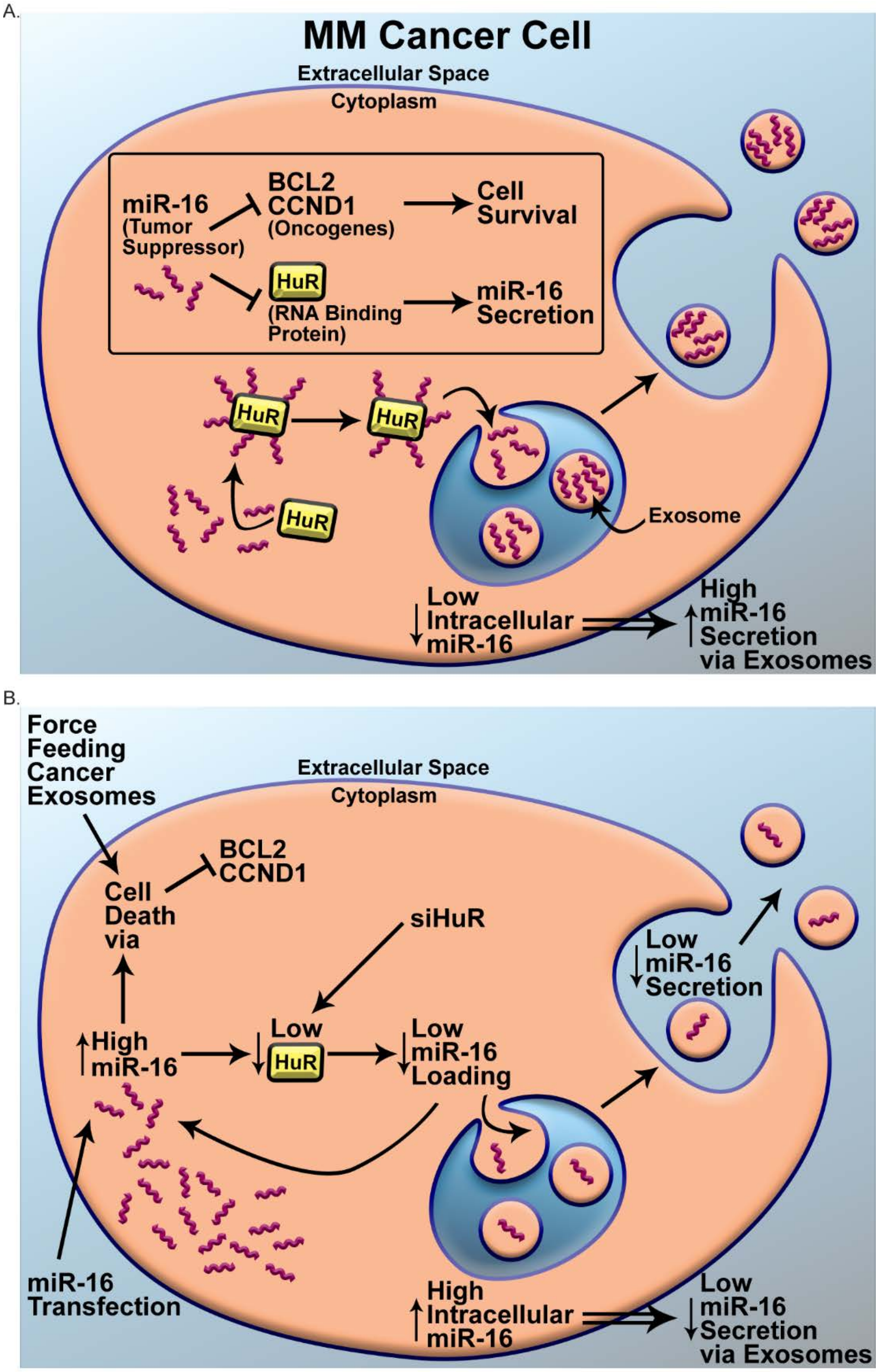

Supplementary Figure 5. Schematic representation of hypothesized mechanism of exosomal miR-16-5p secretion in MM cancer cells. According to our results, along with published literature, we concluded that in malignant mesothelioma (MM) cancer cells A) miR-16-5p is loaded into exosomes, with the help of the RNA binding protein

HuR, and is secreted at high levels in those exosomes. Further, we know that miR-16-5p, a potent tumor suppressor, acts to block gene expression of oncogenic BCL2 and CCND1, and that miR-16-5p negatively regulates HuR, leading to the circumstance that MM cancer cells have low intracellular miR-16-5p and high exosomal miR-16-5p. Our further experiments indicated that: B) force-feeding of MM cancer exosomes to MM cancer cells leads to cell death by blocking oncogenic protein abundances of BCL2 and CCND1; miR-16-5p transfection leads to low HuR levels therefore low miR-16-5p secretion and replenished miR-16-5p intracellular stores, also leading to cell death by BCL2 and CCND1 regulation; and that direct regulation of HuR by siHuR can recapitulate this same cycle of potential therapeutic targeting to increase miR-16-5p expression in cancer cells. Exosome inhibition was omitted from the schematic for clarity and because the role of inhibiting exosome secretion in MM cancer cells on HuR levels has not been elucidated.

Supplementary Table 1. List of differentially expressed miRNAs in MM cancer exosomes compared to non-cancer mesothelial cell exosomes.

Transcript ID(Array Design)	Mesothelioma Avg (log2)	Normal Avg (log2)	Fold Change	P-val
hsa-miR-30a-5p	6.12	2.79	10.05	4.58E-06
hsa-miR-16-5p	6.89	4.18	6.52	0.0015
hsa-miR-92b-3p	4.78	2.4	5.2	0.0257
hsa-miR-1268a	5.76	3.66	4.31	0.0433
hsa-miR-25-3p	4.57	2.52	4.14	0.0361
hsa-miR-320e	5.54	3.57	3.92	4.30E-06
hsa-miR-222-3p	7.37	5.49	3.7	0.0036
hsa-miR-15b-5p	4.26	2.41	3.62	0.0063
hsa-miR-92a-3p	9.96	8.13	3.56	0.0185
hsa-miR-320d	7.71	5.96	3.36	0.0009

hsa-miR-320c	9.46	7.8	3.17	0.0095
hsa-miR-20a-5p	5	3.47	2.88	0.0191
hsa-miR-320a	9.5	8.02	2.79	0.0035
hsa-miR-320b	9.45	8	2.72	0.0049
hsa-miR-4445-3p	4.06	2.65	2.66	0.0231
hsa-miR-7114-5p	2.77	1.39	2.6	0.0246
hsa-miR-548ap-3p	3.36	2.17	2.28	0.0206
hsa-mir-7515	4.36	3.31	2.08	0.0447
hsa-miR-744-5p	3.55	2.54	2.02	0.0365
hsa-miR-3910	2.2	1.2	2.01	0.0018
hsa-miR-885-3p	2.63	3.67	-2.05	0.0165
hsa-miR-4655-5p	1.93	2.97	-2.05	0.0203
hsa-miR-6124	1.35	2.4	-2.07	0.0123
hsa-miR-4681	1.67	2.73	-2.09	0.0029
hsa-miR-6820-5p	2.35	3.45	-2.14	0.0178
hsa-miR-5196-5p	1.48	2.6	-2.17	0.004
hsa-miR-6875-5p	1.15	2.27	-2.17	0.0065
hsa-miR-3162-5p	1.47	2.6	-2.18	0.0011
hsa-miR-3619-5p	1.97	3.1	-2.19	0.0314
hsa-miR-6775-5p	4.05	5.2	-2.21	0.0185
hsa-miR-6127	1.69	2.84	-2.23	0.0054
hsa-miR-4707-5p	3.93	5.12	-2.29	0.0027
hsa-miR-6125	5.39	6.61	-2.32	0.0341
hsa-miR-1233-5p	3.35	4.57	-2.32	0.0156
hsa-miR-6729-5p	6.31	7.53	-2.33	0.0026
hsa-miR-1207-5p	3.64	4.86	-2.33	0.0352
hsa-miR-498	3.25	4.48	-2.33	0.0298
hsa-miR-6077	1.69	2.94	-2.38	0.0015
hsa-mir-4281	2.36	3.62	-2.39	8.84E-05
hsa-miR-4787-5p	6.9	8.18	-2.44	0.0014
hsa-miR-135a-3p	1.06	2.35	-2.46	0.0017
hsa-miR-7109-5p	1.56	2.86	-2.47	0.0038
hsa-miR-5787	7.89	9.23	-2.54	0.0054
hsa-miR-210-3p	3.87	5.21	-2.54	0.042
hsa-miR-6724-5p	3.95	5.37	-2.66	0.0123
hsa-mir-6800	3.31	4.73	-2.68	5.50E-05
hsa-miR-6848-5p	2.59	4.02	-2.69	0.0304
hsa-miR-6787-5p	2.95	4.38	-2.7	0.0116
hsa-miR-6782-5p	3.33	4.79	-2.75	0.0049
hsa-miR-4665-5p	1.5	2.95	-2.75	0.006
hsa-miR-6850-5p	3.37	4.84	-2.76	0.0012
hsa-miR-5100	1.56	3.08	-2.87	0.0156

hsa-miR-6791-5p	3.72	5.25	-2.89	0.0026
hsa-miR-204-3p	1.59	3.16	-2.97	0.0181
hsa-miR-4749-5p	2.69	4.33	-3.13	0.0001
hsa-miR-4417	0.83	2.48	-3.14	0.0002
hsa-miR-4758-5p	2.39	4.04	-3.14	0.0138
hsa-miR-6727-5p	5.4	7.11	-3.28	0.0014
hsa-miR-6858-5p	3.49	5.21	-3.28	0.0005
hsa-mir-6800	3.25	4.98	-3.33	4.85E-05
hsa-miR-1227-5p	3.16	4.92	-3.4	0.0026
hsa-miR-4674	1.86	3.63	-3.41	0.0051
hsa-miR-6824-5p	1.57	3.37	-3.48	0.008
hsa-miR-8069	4.97	6.77	-3.49	0.0251
hsa-miR-7108-5p	3.58	5.39	-3.5	0.0116
hsa-miR-1915-3p	4.95	6.76	-3.51	0.0014
hsa-miR-4689	1.3	3.13	-3.55	0.0325
hsa-miR-149-3p	4.62	6.46	-3.59	0.0056
hsa-miR-6786-5p	5.73	7.6	-3.64	0.0018
hsa-miR-1909-3p	1.2	3.07	-3.66	0.0048
hsa-let-7e-5p	2.04	3.96	-3.79	5.73E-05
hsa-miR-4492	2.44	4.37	-3.82	0.0005
hsa-mir-4466	1.92	3.89	-3.93	0.0022
hsa-miR-6891-5p	1.87	3.85	-3.94	0.0126
hsa-miR-3665	6	8	-4	0.0099
hsa-miR-3196	4.67	6.68	-4.01	0.0128
hsa-miR-6743-5p	3.05	5.06	-4.02	0.023
hsa-miR-3960	7.34	9.36	-4.06	0.0025
hsa-miR-6716-5p	1.38	3.41	-4.07	0.0093
hsa-miR-3180-3p	1.91	3.95	-4.1	0.0182
hsa-miR-6805-5p	3.42	5.46	-4.13	0.0285
hsa-miR-6771-5p	1.86	3.91	-4.13	0.0016
hsa-miR-6722-3p	2.14	4.25	-4.33	0.0021
hsa-miR-7110-5p	2.2	4.32	-4.37	0.002
hsa-miR-8072	5.39	7.6	-4.61	0.0036
hsa-miR-3656	4.76	7.04	-4.84	0.0013
hsa-miR-6802-5p	1.21	3.49	-4.84	0.0171
hsa-miR-4651	3.41	5.7	-4.9	0.0007
hsa-miR-6798-5p	3.16	5.51	-5.1	0.0052
hsa-miR-1273g-3p	3.74	6.11	-5.19	0.0105
hsa-miR-4649-5p	2.3	4.68	-5.19	0.001
hsa-miR-6803-5p	5.16	7.57	-5.31	3.29E-05
hsa-miR-1225-5p	1.6	4.01	-5.32	0.0334

hsa-miR-4488	4.71	7.19	-5.54	0.0111
hsa-miR-6869-5p	4.81	7.28	-5.56	0.0025
hsa-miR-3648	1.55	4.08	-5.76	4.26E-05
hsa-miR-3663-3p	1.46	4.04	-5.99	1.64E-05
hsa-miR-4466	5.7	8.33	-6.18	0.0003
hsa-miR-6752-5p	3.61	6.24	-6.19	0.0015
hsa-miR-6088	4.96	7.6	-6.23	0.0001
hsa-miR-4433b-3p	2.4	5.09	-6.44	0.0049
hsa-miR-4508	4.51	7.22	-6.56	6.58E-05
hsa-miR-6087	6.18	8.9	-6.58	5.78E-05
hsa-miR-1228-5p	3.89	6.67	-6.84	0.0003
hsa-miR-6789-5p	1.99	4.77	-6.87	0.0005
hsa-miR-3940-5p	3.09	5.9	-7.03	0.0088
hsa-miR-6749-5p	1.84	4.71	-7.3	0.0036
hsa-miR-4463	2.47	5.4	-7.61	0.0009
hsa-miR-4687-3p	3.02	6.04	-8.13	0.002
hsa-miR-4497	7.03	10.11	-8.45	0.0012
hsa-miR-6765-5p	2.96	6.1	-8.82	0.0003
hsa-miR-6090	6.32	9.5	-9.06	0.0003
hsa-miR-6089	6.5	9.77	-9.63	0.0002
hsa-mir-6089-1	2.4	5.73	-10.1	0.0002
hsa-mir-6089-2	2.4	5.73	-10.1	0.0002
hsa-miR-1343-5p	1.72	5.07	-10.14	3.07E-06
hsa-miR-31-5p	1.32	4.83	-11.4	0.0002
hsa-miR-328-5p	2.98	6.54	-11.81	0.0003
hsa-miR-3197	3.95	7.8	-14.5	0.0285
hsa-miR-1237-5p	4.63	8.53	-14.86	1.97E-05
hsa-miR-762	4.39	8.37	-15.73	4.18E-05
hsa-miR-4745-5p	2.59	6.61	-16.25	0.001
hsa-miR-4516	3.26	7.34	-16.88	0.0003
hsa-miR-6816-5p	3.01	7.58	-23.72	0.0001
hsa-miR-6780b-5p	2.38	7.17	-27.84	2.72E-06
hsa-miR-3178	4.72	9.54	-28.29	0.0012
hsa-miR-4281	1.8	6.94	-35.37	4.75E-06

hsa-miR-4532	1.83	7.14	-39.78	9.00E-07
hsa-miR-6126	4.73	10.36	-49.59	8.07E-05
hsa-miR-4484	3.06	10.06	-128.2	3.05E-06

Full Western Blot Images from Figures.

Supplementary Figures and Table

Western Blot Full Images

Supp. Fig. 1C

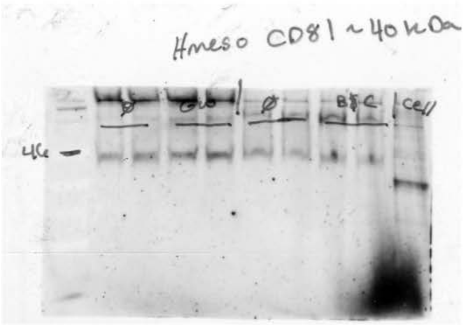

Fig 3D

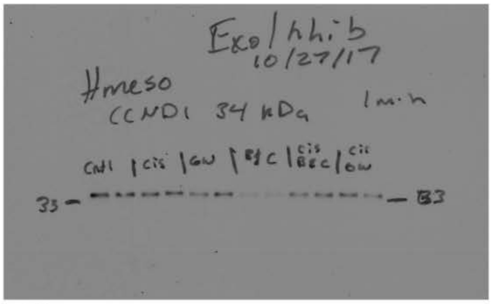

Fig 3D

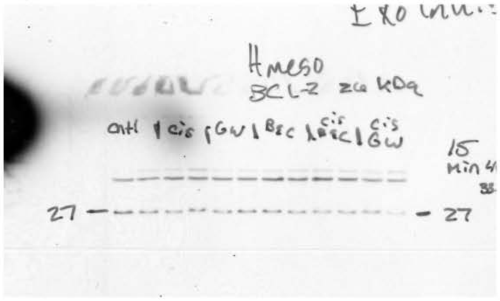

Fig 3E

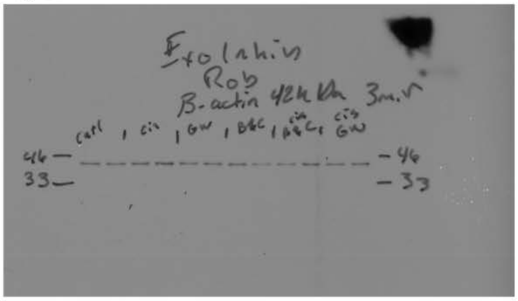

Fig 3D

Fig 3E

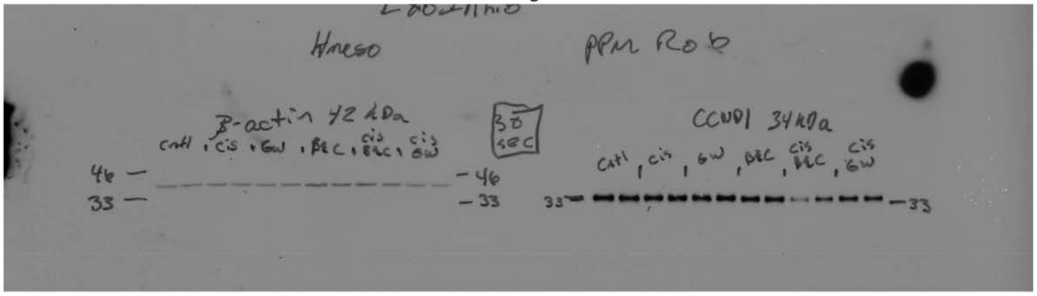

Fig 4H

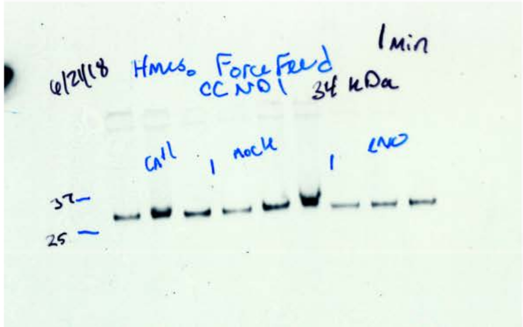

Fig 4H

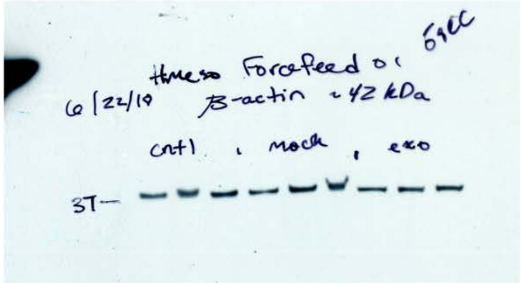

Supplementary Figures and Table

Western Blot Full Images

Fig 5C

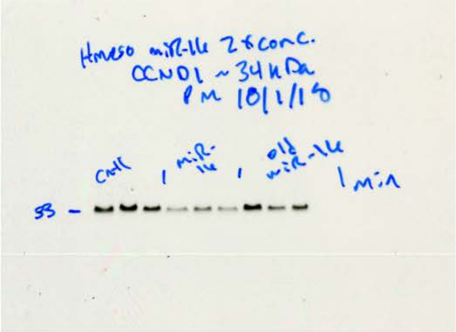

Fig 5C

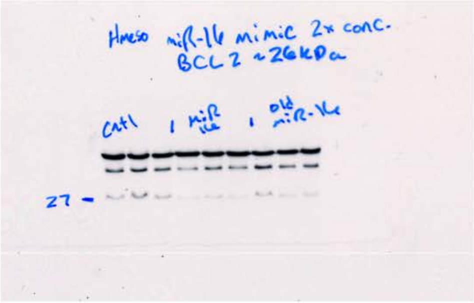

Fig 5C & 6D

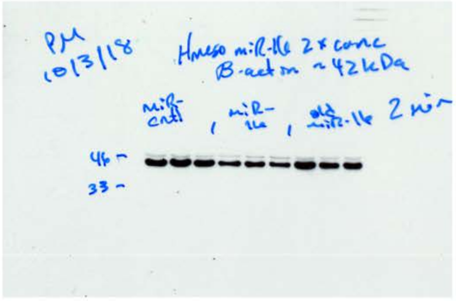

Fig 6D

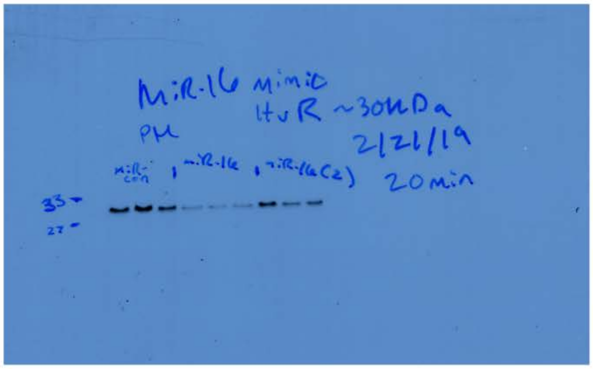

Fig 6A

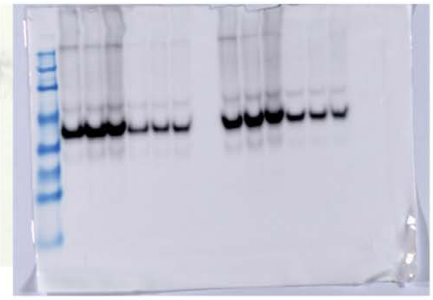

Fig 6A

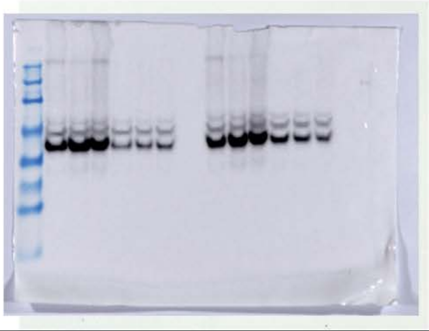

Supplement: Supplementary file 1 — Supplementary Figures [file 41598_2019_48133_MOESM1_ESM.pdf]
